# Potent broadly neutralizing antibodies mediate efficient antibody-dependent phagocytosis of HIV-infected cells

**DOI:** 10.1371/journal.ppat.1012665

**Published:** 2024-10-28

**Authors:** Brian J. Snow, Nida K. Keles, Michael W. Grunst, Sanath Kumar Janaka, Ryan T. Behrens, David T. Evans

**Affiliations:** 1 Department of Pathology and Laboratory Medicine, University of Wisconsin-Madison, Madison, Wisconsin, United States of America; 2 Department of Microbial Pathogenesis, Yale University School of Medicine, New Haven, Connecticut, United States of America; 3 Wisconsin National Primate Research Center, Madison, Wisconsin, United States of America; National Institute for Communicable Diseases, SOUTH AFRICA

## Abstract

Antibody-dependent cellular phagocytosis (ADCP) has been implicated in protection against HIV-1. However, methods for measuring ADCP currently rely on the phagocytosis of gp120- or gp41-coated beads that do not reflect physiologically relevant conformations of the viral envelope glycoprotein or the size of a virus-infected cell. We therefore developed a novel approach for measuring ADCP of HIV-infected cells expressing natural conformations of Env. A monocytic cell line (THP-1 cells) or primary human monocytes were incubated with a CD4+ T cell line that expresses eGFP upon HIV-1 infection in the presence of antibodies and ADCP was measured as the accumulation of eGFP+ material by flow cytometry. The internalization of HIV-infected cells by monocytes was confirmed visually by image-capture flow cytometry. Cytoskeletal remodeling, pseudopod formation and phagocytosis were also observed by confocal microscopy. We found that potent broadly neutralizing antibodies (bnAbs), but not non-neutralizing antibodies (nnAbs), mediate efficient phagocytosis of cells infected with either primary or lab-adapted HIV-1. A nnAb to a CD4-inducible epitope of gp120 (A32) failed to enable ADCP of HIV-infected cells but mediated efficient phagocytosis of gp120-coated beads. Conversely, a bnAb specific to intact Env trimers (PGT145) mediated potent ADCP of HIV-infected cells but did not facilitate the uptake of gp120-coated beads. These results underscore the importance of measuring ADCP of HIV-infected cells expressing physiologically relevant conformations of Env and show that most antibodies that are capable of binding to Env trimers on virions to neutralize virus infectivity are also capable of binding to Env on the surface of virus-infected cells to mediate ADCP.

## Author summary

In addition to neutralizing virus-infectivity, antibodies that bind to the human immunodeficiency virus (HIV-1) envelope glycoprotein (Env) can recruit cells of the immune system to eliminate productively infected cells through a process known as antibody-dependent cellular phagocytosis (ADCP). ADCP occurs when an antibody bound to Env on the surface of an HIV-infected cell is recognized by a phagocytic cell, such as a monocyte, macrophage or neutrophil, and the phagocytic cell engulfs and destroys the virus-infected cell. ADCP has been implicated in protection against HIV-1. However, methods for measuring ADCP currently do not reflect physiologically relevant conformations of Env or the size of a virus-infected cell. We therefore developed a novel assay for measuring ADCP of HIV-infected cells expressing natural conformations of Env. Using this approach, we found that potent broadly neutralizing antibodies (bnAbs) that are capable of binding to functional Env trimers on virions to block HIV-1 infectivity can also mediate efficient phagocytosis of HIV-infected cells. However, non-neutralizing antibodies (nnAbs) that by definition cannot block viral infectivity exhibit little or no ADCP. These results stand in marked contrast to previous methods for measuring ADCP based on the internalization of beads coated with recombinant Env proteins, which show greater antibody-dependent phagocytosis by nnAbs. These results show that most antibodies that are able to neutralize HIV-1 infectivity are also able to direct the phagocytosis of HIV-infected cells and illustrate the importance of measuring ADCP of virus-infected cells expressing physiologically relevant conformations of Env.

## Introduction

Broadly neutralizing antibodies (bnAbs) can protect against simian-human immunodeficiency virus (SHIV) challenge in nonhuman primates and against HIV-1 acquisition in human clinical trials [1–9]. Although most evidence supports neutralization as a critical determinant of antibody-mediated protection [10,11], some studies suggest that the elimination of HIV-infected cells by Fc-dependent effector functions may also contribute to protection [3,12–18]. One of these effector functions, termed antibody-dependent cellular phagocytosis (ADCP), results in the uptake of antibody-opsonized cells through IgG binding to Fcγ receptors on the surface of phagocytic cells such as monocytes and macrophages [19].

Although ADCP was not identified as a correlate of protection in the RV144 trial [16], subsequent characterization of antibodies isolated from vaccinated subjects suggests that ADCP, among other Fc-mediated effector functions, may have contributed to a reduced risk of HIV-1 acquisition [17,18,20–22]. ADCP has also been implicated in protection against SHIV and SIV challenge in nonhuman primate studies [23–27]. However, methods for measuring ADCP have relied on quantifying antibody-mediated phagocytosis of fluorescent beads around 1μm in size coated with recombinant gp120/gp160 or gp41 peptides that do not represent physiologically relevant conformations of the HIV-1 envelope glycoprotein (Env) or the size of a virus-infected cell [13,27,28]. The predominant form of Env on the surface of infected cells and virions is a trimer composed of three gp120 surface subunits non-covalently bound to three gp41 transmembrane subunits [29,30]. Outer domain surfaces of Env are covered by extensive N-linked glycosylation, referred to as the “glycan shield” [31], and the CD4 and co-receptor binding sites are concealed by conformational mechanisms prior to CD4 engagement [32]. Furthermore, antigenic surfaces of gp120 and gp41 involved in gp120-gp120 and gp120-gp41 contacts are inaccessible in the closed Env trimer [32–34]. Env expression on the surface of infected cells is tightly regulated prior to virus assembly by endocytosis motifs in the cytoplasmic tail of gp41 [35–37]. CD4 downmodulation by the viral Nef and Vpu proteins also prevents the exposure of CD4-induced epitopes on virus-infected cells that are frequently targeted by nnAbs [38,39]. Hence, there is little exposure of Env on the surface of HIV-1-infected cells, and the Env that is present is highly resistant to most antibodies. ADCP assays based on the internalization of beads coated with monomeric gp120/gp160 or gp41 do not capture these features of Env and more closely reflects the clearance of viral debris than productively infected cells. Thus, more physiologically relevant methods for measuring ADCP are needed to better define the types of antibodies that are capable of directing the phagocytosis of HIV-1-infected cells.

Although previous studies have investigated antibody-dependent phagocytosis (ADP) of cell-free HIV-1 [40,41], to our knowledge, the phagocytosis of productively infected cells has not been rigorously examined. Moreover, because high viral loads in HIV-infected individuals are sustained by productively infected cells [42], antibody-dependent mechanisms for the elimination of virus-infected cells are likely to have a greater impact on virus replication *in vivo* than the clearance of cell-free virus. We therefore developed a novel flow cytometry assay that is designed to measure ADCP of HIV-1-infected cells. Our assay is based on the internalization of an HIV-infected CD4+ T-cell line by monocytes in the presence of increasing concentrations of antibodies. Using this approach, we show that bnAbs that are capable of binding to functional Env trimers mediate efficient ADCP of HIV-1-infected cells, whereas ADCP by nnAbs is inefficient and does not titer with increasing antibody concentrations.

## Results

### ADCP of HIV-infected cells

**Fig 1:**
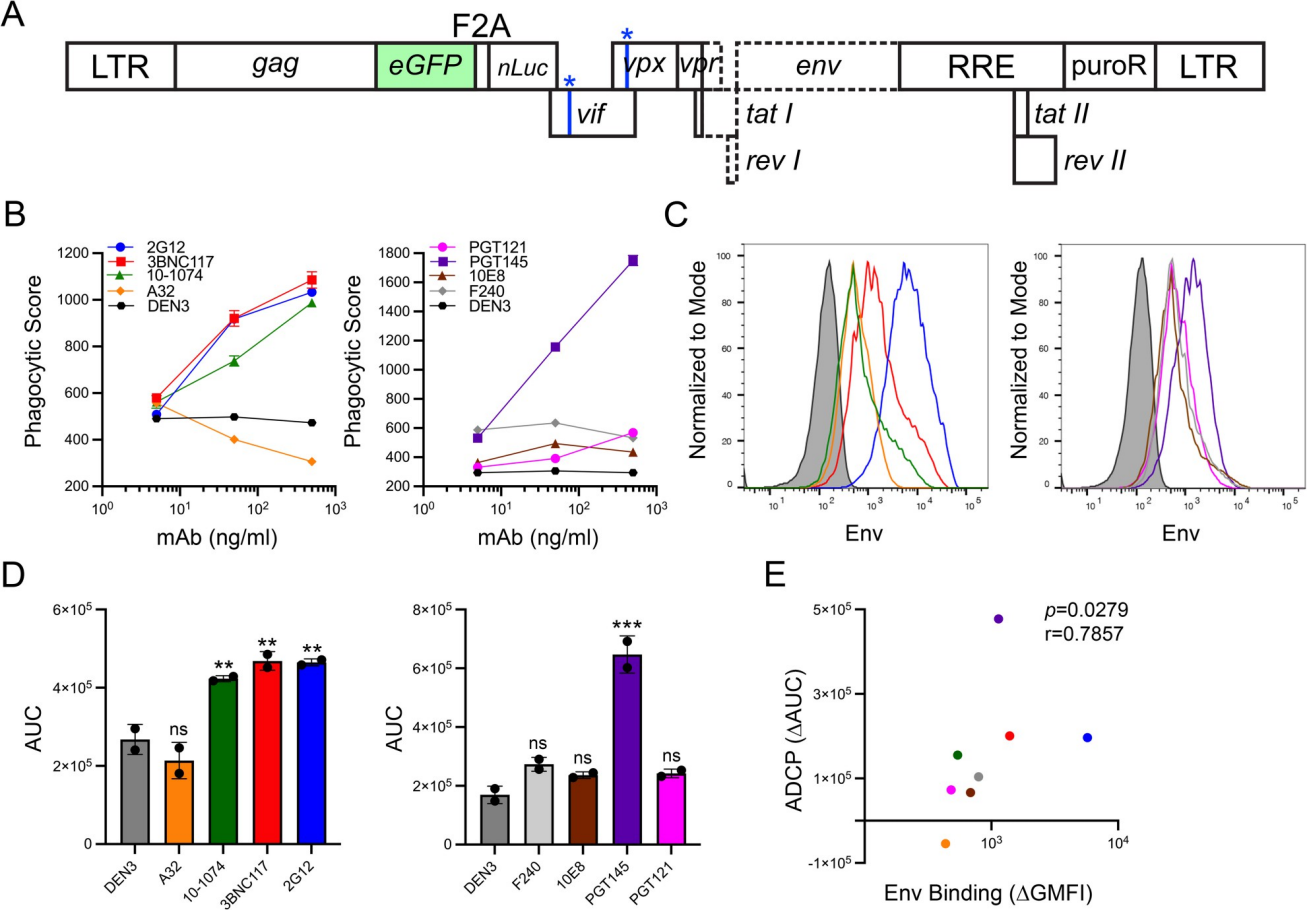
ADCP of HIV-infected cells by a monocytic cell line. (A) SCR84 cells were established by transducing SupT1-CCR5 cells with an SIV_mac_239 vector modified for expression of Gag-eGFP followed by nLuc and puromycin resistance from the *nef* locus. The SIV *vif* and *vpr* genes were inactivated with premature stop codons (asterisks) and the *vpr*, *tat*, *rev* and *env* genes were inactivated with a 1.6 kb deletion (dashed lines). (B) Two days after infection with HIV-1 NL4-3, SCR84 cells were incubated with PKH26-labeled THP-1 cells for 4.5 hours at a 1:3 ET ratio in the presence of the indicated antibodies. Phagocytic scores were calculated by multiplying the percentage of single PKH26+eGFP+ THP-1 cells by the GMFI of eGFP within the THP-1 cells. Error bars represent standard deviation of the mean for triplicate wells. (C) The binding of each antibody to Env on the surface of HIV-1 NL4-3-infected (eGFP+CD4low) SCR84 cells was determined by flow cytometry. Env staining was detected by staining with an AF647-conjugated goat anti-human antibody. (D) Mean area under the curve (AUC) values were calculated from phagocytic scores of duplicate assays. Asterisks denote significant differences by ordinary one-way ANOVA with Dunnett’s test (ns, not significant, *p* > 0.05; *, *p* ≤ 0.05; **, *p* ≤ 0.01; ***, *p* ≤ 0.001). (E) Spearman’s rank-order correlation coefficient was calculated to compare the differences in ADCP (ΔAUC) and Env binding (ΔGMFI) between each of the Env-specific antibodies and the DEN3 control antibody.

To measure ADCP of HIV-infected cells, we established a CD4+ T cell line with an enhanced green fluorescent protein (eGFP) reporter gene that is induced by virus infection. SupT1-CCR5 cells were transduced with an SIV_mac_239 vector encoding Gag-eGFP and nano-Luciferase (nLuc) separated by an F2A ribosomal skip sequence in place of *pol* and a puromycin resistance gene at the *nef* locus (**Fig 1A**). This vector also includes premature stop codons in *vif* and *vpx* and a 1.6 kb deletion spanning *vpr*, the first exons of *tat* and *rev* and the 5’ end of *env*. Thus, all viral genes except *gag* are inactivated, while the *cis*-acting Rev response element (RRE) at the 3’ end *env* is retained to enable Rev-dependent nuclear export of unspliced viral transcripts (**Fig 1A**). Gag-eGFP expression is therefore tightly regulated by both the viral Tat and Rev proteins. Upon HIV-1 or SIV infection of these cells (designated SCR84 cells), expression of Tat and Rev efficiently upregulate Gag-eGFP with minimal background signal.

ADCP is measured as the internalization of HIV-infected (eGFP+) SCR84 cells by the monocytic THP-1 cell line in the presence of increasing concentrations of Env-specific antibodies. THP-1 cells are labeled with the lipophilic dye PKH26 and incubated alone or together with HIV-infected SCR84 cells in the presence of antibody for 4.5 hours at a 1:3 effector-to-target ratio. The percentage of THP-1 cells (PKH26+) that internalize HIV-infected eGFP+ SCR84 cell material and the geometric mean fluorescence intensity (GMFI) of eGFP within the THP-1 cell population is determined by flow cytometry (**S1 Fig**). As a quantitative value that reflects both the frequency of phagocytosis and the amount of material internalized on a per-cell basis, phagocytic scores were calculated by multiplying the percentage of eGFP+PKH26+ THP-1 cells by the GMFI of eGFP within the THP-1 cell population.

Using this approach, monoclonal antibodies specific for diverse epitopes of HIV-1 Env were initially tested for ADCP of SCR84 cells infected with the tier 1 neutralization-sensitive lab-adapted strain HIV-1 NL4-3. These antibodies (all IgG1) included bnAbs to the CD4-binding site (CD4bs) (3BNC117), V3 supersite (10–1074 and PGT121), V2 apex (PGT145), gp120 high mannose glycans (2G12) and gp41 membrane-proximal external region (MPER) (10E8), as well as nnAbs to a CD4-inducible (CD4i) cluster A epitope in gp120 (A32) and a cluster I epitope in gp41 (F240). The dengue virus-specific antibody DEN3 was also included as a negative control. With the exception of PGT121 and 10E8, all of the bnAbs mediated efficient phagocytosis of HIV-1 NL4-3-infected cells (**Fig 1B**). In contrast, the responses for the nnAbs A32 and F240 were low and did not increase with increasing antibody concentrations (**Fig 1B**). The level of Env staining by each antibody on the surface of infected cells also reflected the efficiency of ADCP. The bnAbs with potent ADCP activity (2G12, 3BNC117, 10–1074 and PGT145) showed higher levels of staining than the antibodies that did not mediate ADCP (PGT121, 10E8, A32 and F240) (**Fig 1C**).

For quantitative comparisons of ADCP, area under the curve (AUC) values were calculated from phagocytic scores over the range of antibody concentrations tested. AUC values from replicate assays for each of the Env-specific antibodies were compared to the DEN3 control. This analysis confirmed significant responses for the bnAbs 2G12, 3BNC117, 10–1074 and PGT145 (**Fig 1D**). In accordance with the lack of a dose-response for 10E8 and PGT121 and for the nnAbs A32 and F240, differences in ADCP for these antibodies were not significant (**Fig 1D**). Overall, ADCP correlated with the level of Env staining by the antibodies on the surface of virus-infected cells, even though the magnitude of ADCP was not always commensurate with Env staining for certain bnAbs (**Fig 1E**). The negligible ADCP activity for PGT121 is consistent with the suboptimal epitope for this antibody on HIV-1 NL4-3 Env and with the inability to detect PGT121 neutralization or antibody-dependent cellular cytotoxicity (ADCC) against this virus [43]. The lack of detectable ADCP for 10E8 is also consistent with its specificity for an epitope that is transiently exposed during viral entry [44,45] and with the inability to detect ADCC against HIV-infected cells with this antibody [43]. The absence of detectable ADCP for A32 and F240 can be attributed to the inaccessibility of their epitopes on closed Env trimers expressed on the surface of HIV-infected cells.

### Visualization of ADCP by image-capture flow cytometry

**Fig 2:**
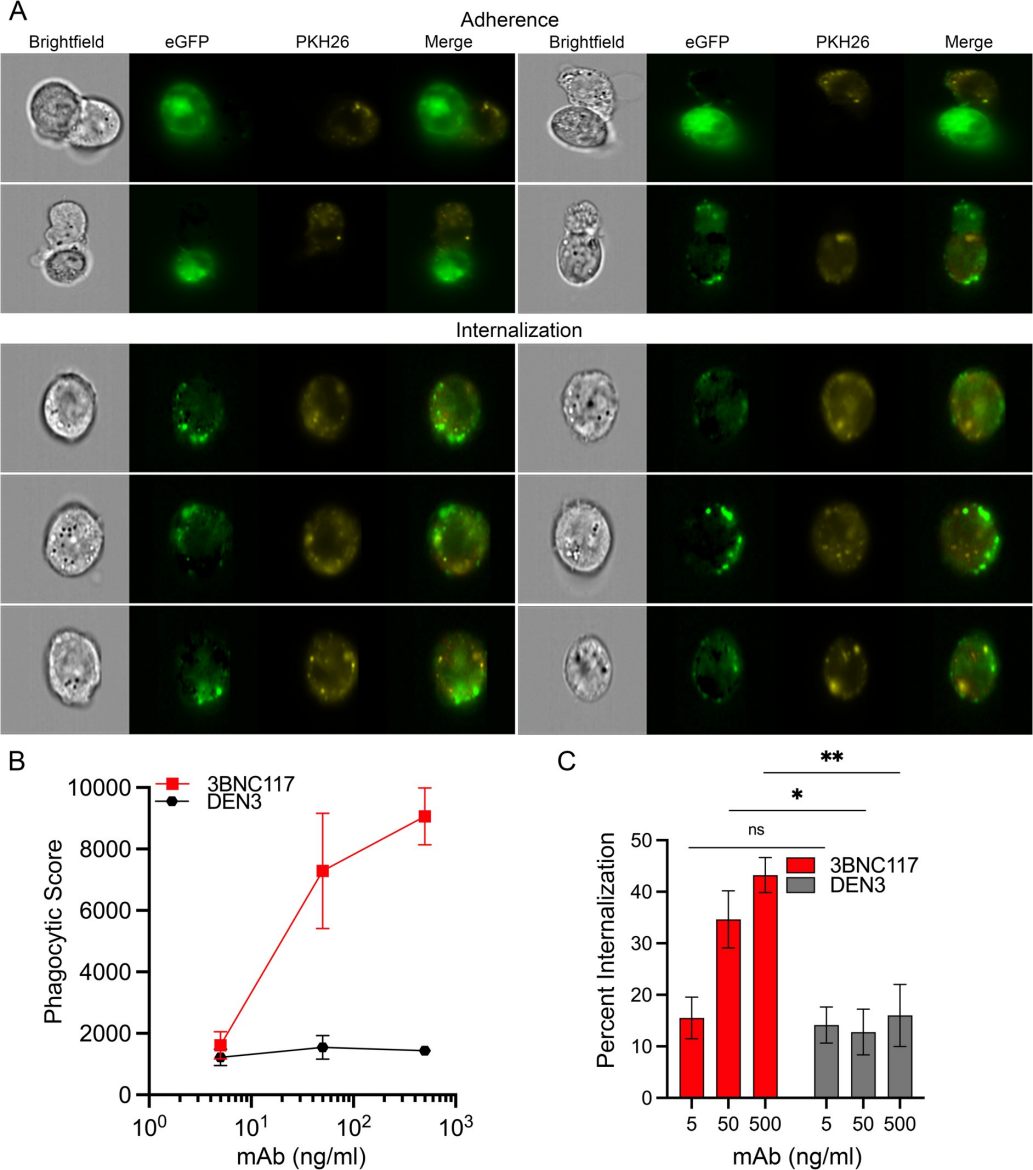
ADCP of HIV-infected cells measured by image-capture flow cytometry. On day 2 post-infection with HIV-1 JR-FL, SCR84 cells were incubated with PKH26-labeled THP-1 effector cells for 4.5 hours at a 1:3 E:T ratio in the presence of the indicated concentrations of 3BNC117 and DEN3. Cells were analyzed using an ImageStream MarkII instrument (Amnis). (A) Representative images were captured showing adherence and phagocytosis of HIV-1-infected (eGFP+) SCR84 cell material by THP-1 (PKH26+) cells. (B) Phagocytic scores were calculated by multiplying the percentage of single PKH26+eGFP+ THP-1 cells by the GMFI of eGFP within the THP-1 cells. (C) Percent internalization was calculated by dividing the percent of PKH26+eGFP+ THP-1 cells with positive internalization scores by the total PKH26+ THP-1 cells as determined using IDEAS software (Amnis version 6.3). Asterisks denote significant differences using paired t tests comparing 3BNC117 to DEN3 at each antibody concentration (ns, not significant, *p* > 0.05; *, *p* ≤ 0.05; **, *p* ≤ 0.01). (B & C) Error bars represent the standard deviation of the mean for triplicate wells.

As visual confirmation of ADCP, the antibody-dependent internalization of HIV-infected cells by THP-1 cells was measured by image-capture flow cytometry. PKH26-labeled THP-1 cells were incubated with HIV-1 JR-FL-infected SCR84 cells in the presence of increasing concentrations of 3BNC117 or DEN3 and data was collected using an ImageStream MarkII instrument. Representative images show the adherence and internalization of eGFP+ material from HIV-infected cells by THP-1 cells (**Fig 2A**). While the adherence of THP-1 cells to HIV-infected cells may represent an intermediate stage of phagocytosis or transient cell-cell contacts, the punctate pattern of eGFP accumulation within the cytoplasm of THP-1 cells clearly indicates phagocytosis of material from HIV-infected cells (**Fig 2A**). Simultaneous assessment of phagocytic scores confirmed ADCP by 3BNC117 (**Fig 2B**). To discriminate between phagocytosed and adherent infected cells, internalization scores were assigned to eGFP+PKH26+ THP-1 cells at each antibody concentration using the internalization feature of the IDEAS image analysis software. THP-1 cells with positive scores representing net eGFP phagocytosis were divided by total single non-adherent THP-1 cells to calculate percent internalization (**Fig 2C**). Whereas the internalization of HIV-infected eGFP+ cells corresponded with increasing 3BNC117 concentrations, there was no increase in the internalization of eGFP+ cells in the presence of DEN3. Image-capture flow cytometry therefore provided visual confirmation of antibody-mediated phagocytosis of HIV-infected cells.

### Time-lapse confocal microscopy reveals dynamic antibody-dependent interactions between monocytic cells and HIV-infected cells

**Fig 3:**
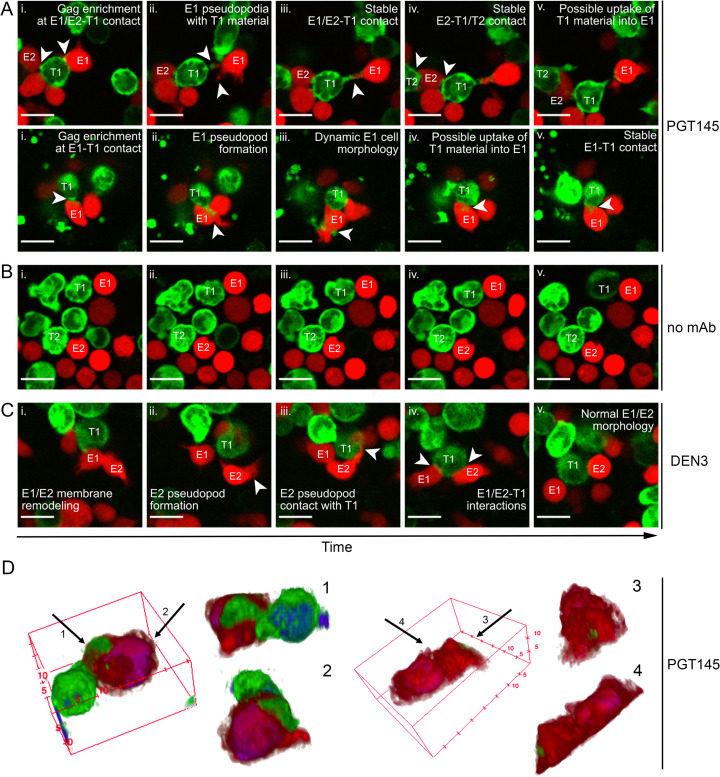
Confocal microscopy capturing time-lapse ADCP and 3D depictions of individual events. Two days following infection with HIV-1 NL4-3, SCR84 cells were co-cultured with mCherry expressing THP-1 cells in the presence of 0.5 μg/ml PGT145 (A), no antibody (B) or DEN3 (C) at a 1:1 E:T ratio. Time-lapse images were captured under normal culture conditions in a microscope stage-mounted live-cell culture chamber at 1-minute intervals over 4 hours. “E” indicates effector cell while “T” indicates target cell. Roman numerals (i-v) indicate captured images in a time course spanning 45–90 minutes. Scale bars represent 20 μm. (D) Co-cultures of HIV-1 NL4-3-infected SCR84 cells (green) and mCherry expressing THP-1 cells (red) were fixed and Hoechst stained (blue) after 4.5 hours in the presence of 0.5 μg/ml of PGT145. Z-stack images were captured at 0.4 μm intervals. Arrows (1–4) indicate rotation of the viewing plane. (Left) A THP-1 cell (red) engulfing a large portion of the cytoplasm of an HIV-infected cell (green). (Right) A THP-1 cell engulfing a small fragment of an HIV-infected cell (green). (A–D) Images were processed and analyzed using FIJI/ImageJ.

Time-lapse confocal microscopy was employed to interrogate the dynamics of antibody-dependent internalization of HIV-infected cells. mCherry-labeled THP-1 cells were incubated with HIV-1 NL4-3-infected SCR84 cells in the presence of PGT145, DEN3 or without antibody. Image capture occurred approximately every minute over 4.5 hours and was initiated within 15 minutes of co-culture. PGT145 increased interactions between THP-1 and HIV-infected cells, which was evident at the earliest time points. Visual indications of ADCP were observed, including membrane remodeling, pseudopod formation, prolonged interactions between effector and target cells and internalization of virus-infected eGFP+ cell material (**Fig 3A**). In contrast, co-cultures in the absence of antibody exhibited few intercellular interactions and little uptake of infected cell material (**Fig 3B**). In the presence of the control antibody DEN3, the THP-1 cells underwent membrane remodeling similar to co-cultures with PGT145 (**Fig 3C**), but exhibited little internalization of infected cell material emphasizing the necessity of Env-binding for ADCP.

To visualize phagocytosis in greater detail, high-resolution Z-stack images of THP-1 cells engaged with HIV-infected cells were captured and reconstructed into 3D models. Images showing THP-1 cell engulfment of a large portion of the cytoplasm of an eGFP+ HIV-infected cell and a smaller piece of eGFP+ cell debris are shown (**Fig 3D**). Together, time-lapse and fixed-cell microscopy reveal effector-target cell dynamics and the requirement for Env-specific antibodies for these interactions. These images further suggest that ADCP of HIV-infected cells of a similar size to THP-1 cells or primary monocytes may occur by sequentially engulfing large fragments of the cytoplasm, ultimately leading to the elimination of infected cells.

### ADCP of cells infected with primary HIV-1 isolates

**Fig 4:**
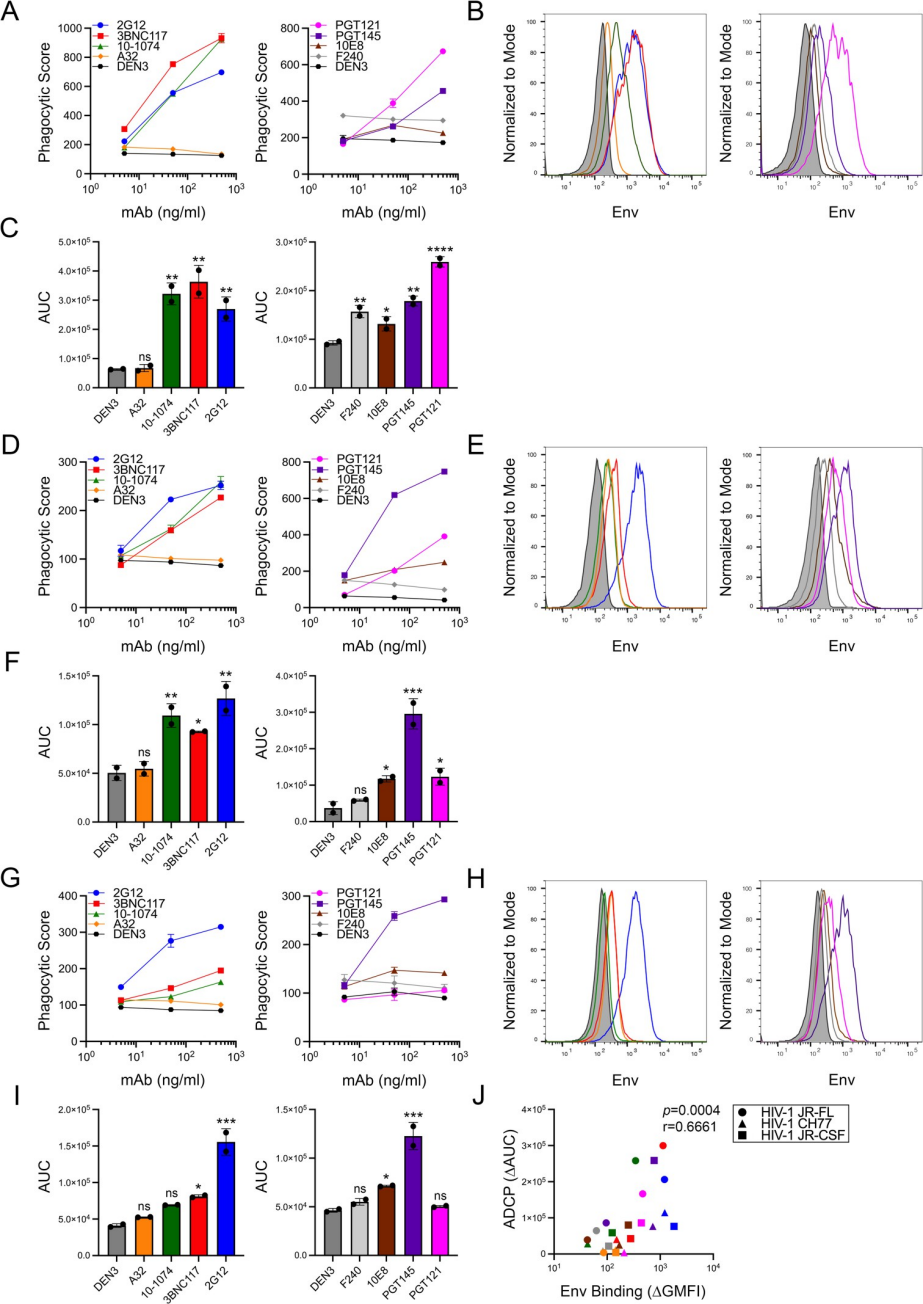
ADCP of cells infected with primary HIV-1 isolates. ADCP and surface expression of Env were measured for cells infected with HIV-1 JR-FL (A & B), JR-CSF (D & E) and CH77 (G & H) by flow cytometry as described in Fig 1. Error bars represent the standard deviation of the mean for triplicate wells. (C, F & I) Mean AUC values were calculated from the phagocytic scores of duplicate assays and compared to the DEN3 control by ordinary one-way ANOVA with Dunnett’s test (ns, not significant, *p* > 0.05; *, *p* ≤ 0.05; **, *p* ≤ 0.01; ***, *p* ≤ 0.001; ****, *p* ≤ 0.0001). (J) Spearman’s rank-order correlation coefficient was calculated to compare the differences in ADCP (ΔAUC) and Env binding (ΔGMFI) between each of the Env-specific antibodies and the DEN3 control antibody.

To assess ADCP against viruses expressing tier 2 neutralization-resistant envelope glycoproteins typical of naturally transmitted HIV-1 field isolates, SCR84 cells were infected with HIV-1 JR-FL, HIV-1 JR-CSF and the transmitted-founder virus HIV-1 CH77. Using the same antibodies that were tested against HIV-1 NL4-3, we found that the bnAbs 2G12, 3BNC117, 10–1074 and PGT145 generally mediated potent ADCP of cells infected with these viruses, whereas the nnAbs A32 and F240 did not (**Fig 4**). However, a few strain-specific differences were observed. Whereas PGT121 directed the phagocytosis of cells infected with HIV-1 JR-FL and HIV-1 JR-CSF (**Fig 4A–4F**), this antibody failed to mediate detectable ADCP of cells infected with HIV-1 CH77 (**Fig 4G–4I**). Furthermore, ADCP was undetectable against any of the viruses for A32, and although slightly elevated phagocytic scores were observed for F240, this response was only significant for HIV-1 JR-FL (**Fig 4C**). ADCP also correlated with antibody binding to Env on the surface of virus-infected cells (**Fig 4J**). Similar to lab-adapted HIV-1 NL4-3, this correlation is primarily driven by consistently low levels of Env staining and ADCP for the nnAbs despite the more variable relationship between Env staining and ADCP among the bnAbs. Thus, ADCP of cells infected with tier 2 neutralization-resistant HIV-1 is most efficient for bnAbs that are capable of binding to closed Env trimers on the surface of virions and infected cells.

### ADCP of HIV-infected cells by primary human monocytes

**Fig 5:**
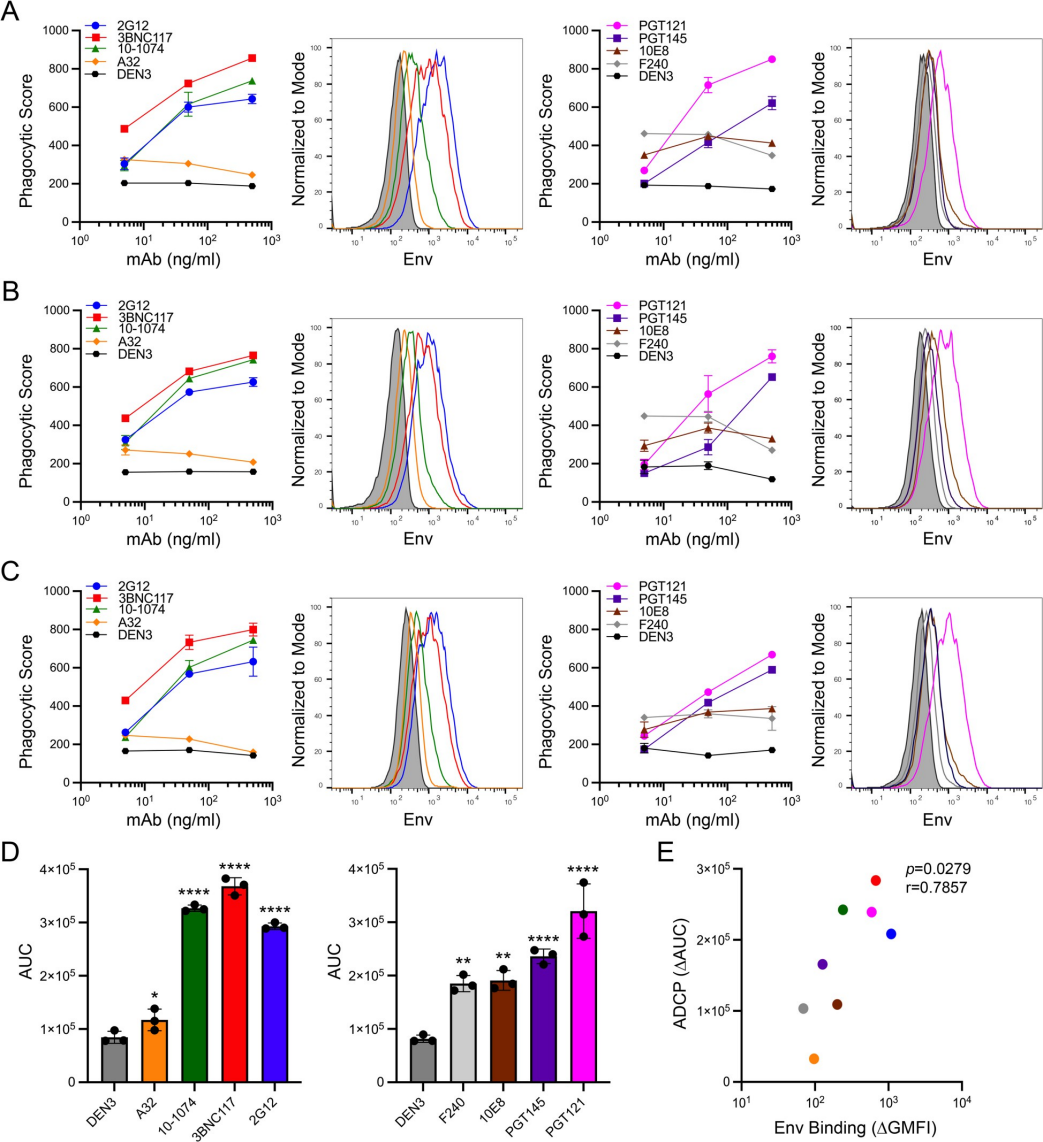
ADCP of HIV-infected cells by primary monocytes. ADCP of HIV-1 JR-FL-infected cells by primary human monocytes from three different donors and surface expression of Env on virus-infected cells (A-C) were measured by flow cytometry as described in Fig 1. Error bars represent the standard deviation of the mean for triplicate wells. (D) Mean AUC values were calculated from the phagocytic scores of these replicate assays and compared to the DEN3 control by ordinary one-way ANOVA with Dunnett’s test (ns, not significant, *p* > 0.05; *, *p* ≤ 0.05; **, *p* ≤ 0.01; ***, *p* ≤ 0.001; ****, *p* ≤ 0.0001). (E) Spearman’s rank-order correlation coefficient was calculated to compare the differences in ADCP (ΔAUC) and Env binding (ΔGMFI) between each of the Env-specific antibodies and the DEN3 control antibody.

ADCP of HIV-infected cells was also measured using primary human monocytes. Classical CD14+CD16- monocytes were isolated from the peripheral blood of three donors and incubated with HIV-1 JR-FL-infected SCR84 cells. In each case, the bnAbs 2G12, 3BNC117, 10–1074, PGT121 and PGT145 mediated potent ADCP that titered with increasing antibody concentrations (**Fig 5A–5C**). However, responses for 10E8 and the nnAbs A32 and F240 were low and did not increase at higher antibody concentrations (**Fig 5A–5C**). Comparisons with the DEN3 control confirmed significant differences in ADCP for 2G12, 3BNC117, 10–1074, PGT121 and PGT145, and weak but detectable responses for 10E8, A32 and F240 (**Fig 5D).** ADCP also correlated with antibody binding to Env on the surface of virus-infected cells (**Fig 5E**). Primary monocytes therefore exhibited very similar ADCP responses as THP-1 cells for each of the Env-specific antibodies.

The ADCP activity of F240 was further investigated since this antibody mediated low but reproducible responses to HIV-1 JR-FL-infected cells with both THP-1 cells and primary monocytes. When ADCP was measured against cells infected with a variant containing a D368A substitution in Env that interferes with CD4 binding [38,46], the ADCP activity of F240 was greatly diminished and no longer distinguishable from the DEN3 control (**S2 Fig**). Thus, some exposure of the epitope for F240 on the post-fusion conformation of gp41 may occur through CD4-binding and the formation of syncytia with uninfected SCR84 cells. This could account for a low level of ADCP activity of F240, which appears to be somewhat more pronounced for HIV-1 JR-FL.

### Comparison of ADCP of HIV-1-infected cells and Env-conjugated beads

**Fig 6:**
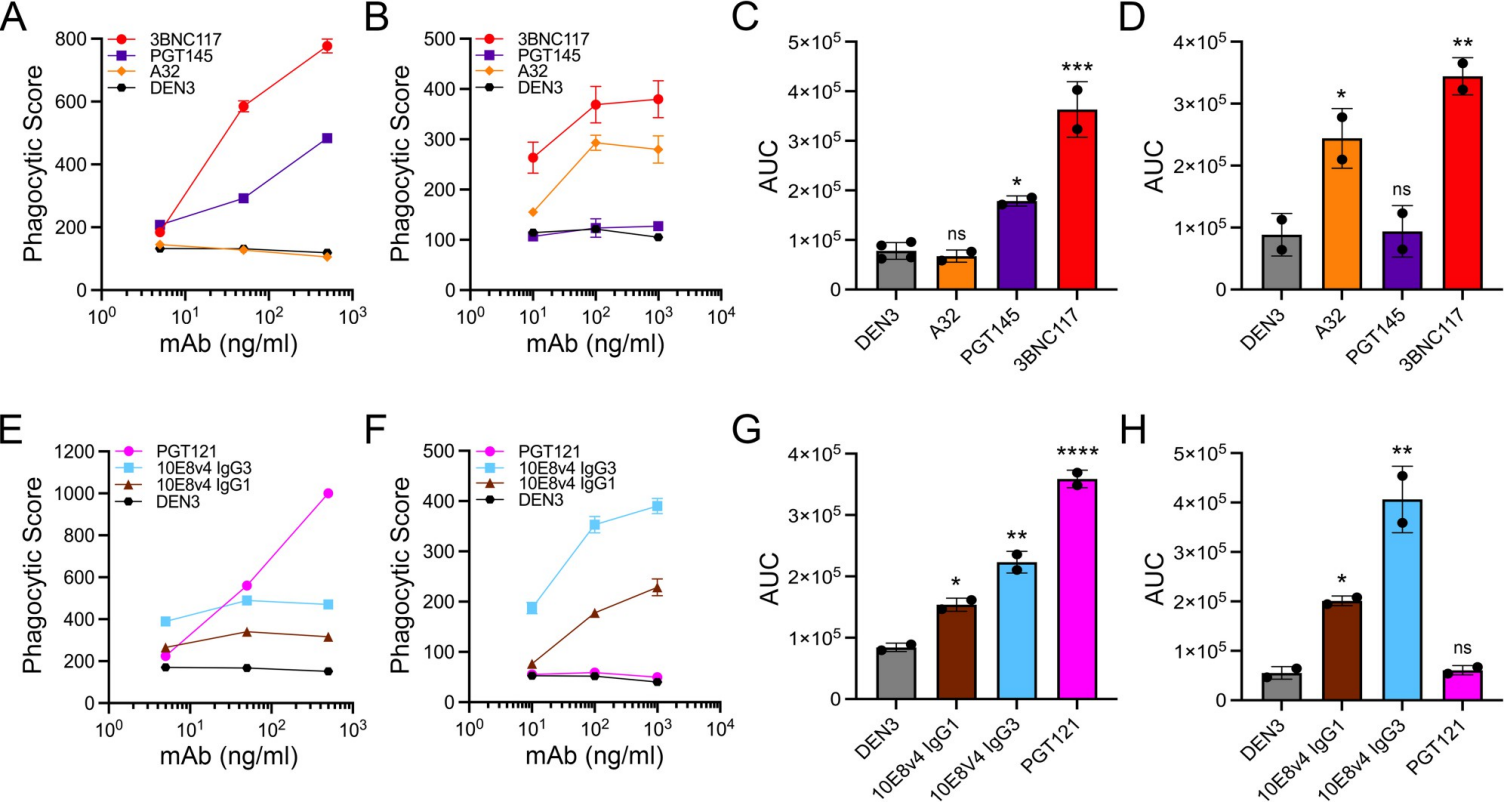
ADCP of HIV-1-infected cells and fluorescent beads coated with monomeric gp120 or gp41 peptide. (A & E) ADCP of HIV-1 JR-FL-infected cells was measured by flow cytometry as described in Fig 1. ADCP of beads was measured by incubating THP-1 cells overnight with fluorescent microspheres coated with monomeric gp120 (B) or gp41 MPER peptide (F) in the presence of the indicated antibodies. Phagocytic scores were calculated by multiplying the percentage of single fluorescent THP-1 cells by the MFI within THP-1 cells and dividing by 1000. Error bars represent the standard deviation of the mean for triplicate wells. Mean AUC values were calculated from duplicate assays with HIV-infected cells (C & G), gp120-coated beads (D) and gp41 MPER peptide-coated beads (H). AUC differences were compared to the DEN3 control by ordinary one-way ANOVA with Dunnett’s test (ns, not significant, *p* > 0.05; *, *p* ≤ 0.05; **, *p* ≤ 0.01; ***, *p* ≤ 0.001; ****, *p* ≤ 0.0001).

Fluorescent microspheres conjugated with monomeric gp120/gp160 or gp41 peptides are routinely used to measure antibody-dependent phagocytosis [27,28]. Env-specific antibodies were therefore compared for their ability to direct the phagocytosis of gp120-conjugated beads and HIV-1-infected cells. In accordance with the exposure of the CD4bs epitope for 3BNC117 on Env trimers and monomeric gp120, this bnAb mediated the phagocytosis of both HIV-1 JR-FL-infected cells and gp120-coated beads (**Fig 6A and 6B**). In contrast, PGT145 only mediated ADCP of virus-infected cells (**Fig 6A and 6B**). Conversely, A32 mediated the phagocytosis of gp120-coated beads (**Fig 6B**) but not HIV-1-infected cells (**Fig 6A**). These observations are supported by comparisons of data from replicate assays showing differences in the internalization of HIV-infected cells and gp120-coated beads (**Fig 6C and 6D**). The inability of PGT145 to mediate the uptake of gp120-coated beads reflects the specificity of this bnAb for a quaternary epitope at the V2-apex of Env trimers that is not present in monomeric gp120 [47]. Likewise, the phagocytic activity of A32 can be attributed to the exposure of the epitope for this nnAb on monomeric gp120, but not on closed Env timers expressed on the surface of virus-infected cells where it is occluded by oligomerization prior to CD4 engagement [38,48].

Similar differences were observed in the ADCP of HIV-1-infected cells and gp41 peptide-conjugated beads. 10E8v4 IgG1, a version of the MPER-specific bnAb 10E8 with increased solubility [49], mediated phagocytosis of beads coated with gp41 peptides that titered with increasing antibody concentrations (**Fig 6F**), but exhibited a much more modest response against cells infected with HIV-1 JR-FL (**Fig 6E**). We hypothesized that the weaker response to virus-infected cells may be due to the suboptimal orientation of 10E8v4 IgG1 when bound to the MPER at the base of gp41 [50], which could limit the accessibility of its Fc domain for Fcγ receptor engagement. We therefore tested an IgG3 version of 10E8v4 with an extended flexible hinge region to facilitate Fcγ receptor binding. Changing the subclass of this antibody to IgG3 increased the phagocytosis of both virus-infected cells and gp41 peptide-coated beads, similar to the effects of IgG3 observed previously by bead-based assays [51] (**Fig 6E and 6F**). However, this effect was much greater for gp41 peptide-coated beads (**Fig 6H**) than HIV-infected cells (**Fig 6G**), indicating better accessibility of the MPER epitope on peptide-coated beads than on virus-infected cells. The use of fluorescent beads conjugated with gp41 peptides may therefore overestimate the magnitude of ADCP responses by gp41 MPER antibodies.

## Discussion

Using a novel approach for measuring antibody-dependent cellular phagocytosis of HIV-infected cells, we found that bnAbs, which are capable of binding to functional Env trimers on the surface of virions to neutralize viral infectivity, are also capable of binding to Env on the surface of infected cells to mediate ADCP. In contrast, nnAbs that by definition cannot block HIV-1 infectivity exhibit little or no ADCP of virus-infected cells. Our results stand in marked contrast to commonly used antibody-dependent phagocytosis assays based on the internalization of fluorescent beads coated with monomeric gp120 or gp41 peptides that show greater ADP by nnAbs.

ADCP of HIV-infected cells was confirmed visually by image-capture flow cytometry and confocal microscopy. The dose-dependent increase in the accumulation of eGFP+ material from HIV-infected SCR84 cells by THP-1 cells in the presence of increasing concentrations of bnAb is consistent with antibody-mediated phagocytosis. Moreover, the punctate pattern of eGFP accumulation within the cell distinguishes phagocytosis from trogocytosis, which is characterized by the rapid exchange of plasma membrane contents through cell-to-cell contact [52]. Time-lapse imaging further revealed that the mechanism of ADCP involves the stimulation of cytoskeletal rearrangements, pseudopod formation and the internalization of material from virus-infected cells. Although monocytes can phagocytose objects larger than themselves, the extent to which cells can adjust their morphology is limited [53,54]. Attempts to phagocytose cells of a similar size, may lead to “frustrated phagocytosis” in which partial engulfment occurs [55,56]. Three-dimensional image reconstruction of THP-1 cells engaged with HIV-infected cells suggests that monocytes can phagocytose virus-infected cells of a similar size by tearing off cytoplasmic fragments, ultimately leading to the destruction and elimination of infected cells.

While ADCP of productively infected cells has not been examined in a systematic way, previous studies have investigated antibody-dependent phagocytosis of cell-free HIV-1. An initial report found that neutralizing antibodies can mediate antibody-dependent virion phagocytosis (ADVP) [40]. However, a later study concluded that many bnAbs poorly mediate ADVP because of the low density of Env on virus particles [41], which is estimated to be as few as 8–14 spikes per virion [57,58]. These differing conclusions can be attributed to differences in the methods for measuring ADVP, as the former study involved spinoculation of monocytes with virus and antibody [40], while the latter study did not and only observed significant phagocytosis of viral aggregates [41]. Env expression on the surface of HIV-infected cells is also tightly regulated prior to the assembly and release of virus particles [59,60], but is nevertheless more abundant on cells than on virions. The greater abundance of Env on the surface of HIV-infected cells creates an opportunity for Fcγ receptor crosslinking and antibody-dependent phagocytosis.

ADCP may also have a greater effect on HIV-1 replication in chronically infected individuals than ADVP. The high levels of viremia characteristic of untreated HIV-1 infection are sustained by continuous rounds of infection and turnover of CD4+ lymphocytes [42]. While the half-life of cell-free HIV-1 in blood is only a few minutes (13 to 26 minutes following HIV-1 infusion into naïve rhesus macaques [61]), the half-life of productively infected cells is approximately 1–2 days [42,62]. The amount of virus produced by an infected cell, known as the viral burst size, is estimated to be at least 5 x 10^4^ virions per cell [63]. Although neutralizing antibodies can reduce the half-life of virus in circulation by a few minutes [61], the elimination of productively infected cells by Fc-mediated mechanisms such as ADCP is therefore expected to have a greater impact on viral loads in HIV-infected individuals than the accelerated clearance of cell-free virus.

All of the bnAbs tested mediated efficient phagocytosis of HIV-infected cells except 10E8. 10E8 exhibited lower levels of Env staining and less ADCP than the other bnAbs. This is consistent with transient exposure of the MPER epitope for this antibody on gp41 [44,45,50] and with previous studies by our group showing that MPER antibodies do not mediate ADCC against virus-infected cells [43,64]. The location of the MPER epitope at the base of gp41 also suggests that the suboptimal orientation of the Fc domain of 10E8 for Fcγ receptor engagement may have contributed to weaker ADCP. Changing the subclass of 10E8v4 to IgG3, which has an extended flexible hinge region, nearly doubled phagocytic scores but did not lead to responses typical of the other bnAbs. However, the IgG3 version of 10E8v4 conferred a much greater increase in the phagocytosis of fluorescent beads coated with gp41 peptides. These observations imply that the MPER epitope is poorly accessible on the surface of HIV-infected cells and that the greater accessibility of this epitope on peptide-conjugated beads overestimates the efficiency of ADCP by this class of antibodies.

Although the ultimate value of an *in vitro* assay is to predict activity *in vivo*, ADCP alone is unlikely to correlate with protection. A meta-analysis of passive antibody transfer experiments in macaques found that serum ID50 titer is predictive of protection against SHIV challenge [10]. However, this does not necessarily mean that neutralization is the only mechanism of protection. Indeed, substitutions in the Fc domain that prevent FcγR-binding impair protection by b12 [3,14]. Other studies also support a role for non-neutralizing effector functions in protective immunity [65–67]. Furthermore, we found that an antibody that binds to Env with insufficient affinity to neutralize viral infectivity can still direct the killing of virus-infected cells by ADCC [68]. ADCP may therefore contribute to antibody-mediated control of virus replication, even if ADCP alone is not sufficient for protection. Thus, measurement of ADCP provides an important indication of the capacity of an antibody to direct the phagocytic uptake of HIV-infected cells, which together with neutralization and ADCC, builds a more complete picture of the range of antiviral antibody functions *in vivo*.

In summary, we have developed a novel approach for measuring antibody-dependent phagocytosis of HIV-infected cells expressing natural conformations of Env. Using this approach, we show that antibodies that are capable of binding to native Env trimers on virions to block viral infectivity are generally capable of directing efficient ADCP of HIV-infected cells. In contrast, nnAbs that cannot bind to the prefusion conformation of Env on virions are poor mediators of the phagocytic uptake of virus-infected cells. These results illustrate the importance of measuring ADCP of HIV-infected cells expressing physiologically relevant conformations of Env.

## Materials and methods

### Plasmids and viral clones

2G12 heavy chain (HC) and light chain (LC) expression plasmids were provided by Dr. Pamela Bjorkman (California Institute of Technology, Pasadena, CA). 3BNC117 and 10–1074 HC and LC expression plasmids were provided by Michel Nussenzweig (Rockefeller University, New York, NY). A32 HC and LC expression plasmids were provided by James Robinson (Tulane University, New Orleans, LA). 10E8v4 IgG1 HC and LC expression plasmids were provided by Peter Kwong (National Institutes of Health, Bethesda, MD). 10E8v4 IgG3 HC expression plasmid was created by using standard cloning methods to replace the HC variable region of the 10E8 IgG3 HC plasmid provided by Peter Kwong with the 10E8v4 HC variable region. Plasmids for the HIV-1 NL4-3, JR-CSF and CH77 infectious molecular clones (IMCs) were obtained through the NIH HIV Reagent Program, Division of AIDS, NIAID, NIH, contributed by Malcolm Martin [69], Irvin S. Y. Chen and Yoshio Koyanagi [70] and John Kappes and Christina Ochsenbauer [71], respectively. A plasmid for the HIV-1 JR-FL IMC was provided by Dennis Burton (The Scripps Research Institute, La Jolla, CA).

The SIV_mac_239-based vector for transduction of Sup T1-CCR5 cells was created as follows. *Pol* was replaced with sequences encoding eGFP and secreted nLuc separated by an F2A ribosomal skip sequence, while retaining the central polypurine tract. A 1.6 kb region spanning the 3’ end of *vpr*, the first exons of *tat* and *rev*, and the 5’ half of *env* was deleted. Premature stop codons were introduced into *vpx* and *vif*, and *nef* was replaced with the *pac* gene for puromycin resistance.

### Monoclonal antibodies

PGT121, 10E8 and F240 were obtained through the NIH HIV Reagent Program, Division of AIDS, NIAID, NIH, contributed by the International AIDS Vaccine Initiative [72], Mark Connors [73], and Marshal Posner and Lisa Cavacini [74], respectively. PGT145 was produced by Catalent Pharma Solutions (Madison, WI) and DEN3 was produced at The Scripps Research Institute (La Jolla, CA).

3BNC117, 10–1074, A32, 2G12, 10E8v4 IgG1 and 10E8v4 IgG3 were produced using the Expi293 expression system (Gibco). Expi293 cells (~9 x 10^7^ cells) were co-transfected with 15 μg of both heavy and light chain antibody expression plasmids using ExpiFectamine 293 reagent (Gibco) in 30 ml cultures. Six days post-transfection, cell culture supernatants were harvested, clarified by centrifugation and filtered using 0.45 μm PES syringe filter discs (Thermo Scientific). IgG1 and IgG3 antibodies were purified using protein A and G Gravitrap columns, respectively (GE Healthcare Life Sciences). Bound IgG1 was eluted into 0.2 ml 1 M sodium carbonate (pH 9.3) by the addition of 3 ml 25 mM sodium phosphate/citrate buffer (pH 3.0) and bound IgG3 was eluted into 0.3 ml 1 M sodium carbonate (pH 9.3) by the addition of 3 ml 25 mM sodium phosphate/citrate buffer (pH 2.5).

### Cell lines

The SCR84 cell line was established by transduction of Sup T1-CCR5 cells with an SIV_mac_239-based vector for Tat-and Rev-inducible expression of Gag-eGFP. The vector was packaged by co-transfecting 293T cells with the modified SIV_mac_239 genome described above, psPAX2, pcRev, pCEP4-SIV Tat and pVSV-G using GenJet In Vitro DNA Transfection Reagent (Ver. II). Following transduction, puromycin-resistant cells were cloned by limiting dilution. THP-1 cells that constitutively express mCherry were established by transduction of parental THP-1 cells with a VSV-G pseudotyped murine leukemia virus (MLV) produced by co-transfecting 293T cells with pCMS28_mCherry (MIGR1-derived retroviral vector), pMD MLV GagPol and pVSV-G using GenJet In Vitro DNA Transfection Reagent (Ver. II). Parental THP-1 cells and primary monocytes were cultured in RPMI medium supplemented with 10% FBS, 2 mM L-Glutamine, 100 U/ml penicillin G, 100 μg/ml streptomycin, and 0.25 μg/ml amphotericin B (R10 medium). SCR84 cells and mCherry-transduced THP-1 cells were maintained in R10 with 0.4 μg/ml and 1 μg/ml puromycin, respectively.

### ADCP assay

SCR84 cells were infected with VSV G-pseudotyped HIV-1 Δ*vif* by spinoculation [75] for 2 hours at 1200 x g in the presence of 40 μg/ml of Polybrene to achieve 60–90% infection when the ADCP assay is performed 2 days later. Four hours post-infection, 100 nM TAK-779 (Tocris Bioscience) was added to cultures infected with CCR5 (R5)-tropic HIV-1 to inhibit syncytia formation. Forty-eight hours post-infection, THP-1 effector cells were stained with 2 μM PKH26 (Millipore Sigma) for 3.5 minutes. THP-1 cells and HIV-infected SCR84 cells were co-incubated at an E:T ratio of 1:3 (5 x 10^4^ cells: 1.5 x 10^5^ cells) in the presence or absence of serial dilutions of antibody for 4.5 hours at 37°C in triplicate wells of round-bottom 96-well plates at 200 μl/well. Following incubation, the cells were washed with 2% FACS buffer (PBS with 2% FBS) and fixed in 2% paraformaldehyde PBS. The percentage of single eGFP+PKH26+ THP-1 cells and the GMFI of eGFP within the PKH26+ THP-1 cell population was determined by flow cytometry using a BD FACSymphony A3 instrument. Phagocytic score was calculated by multiplying the percentage of eGFP+PKH26+ cells by the GMFI of eGFP within the PKH26+ cell population.

For ADCP assays using primary monocytes, peripheral blood was collected by venipuncture from three blood donors using K2 EDTA Vacutainer tubes (BD Biosciences) in accordance with protocol 2015–0900 approved by the University of Wisconsin-Madison Institutional Review Board. Within 1 hour of collection, peripheral blood mononuclear cells (PBMCs) were separated from whole blood by centrifugation over Ficoll-Paque PLUS (Cytiva). Classical monocytes (CD14++CD16-) were isolated from PBMCs using the Classical Monocyte Isolation Kit (Miltenyi Biotec).

### Env staining

HIV-infected SCR84 cells were stained with near-IR fluorescent amine-reactive dye (Invitrogen, Ref: L34976A) to distinguish live and dead cell populations and with 10 μg/ml of bnAbs and nnAbs for surface expression of Env. The binding of these Env-specific monoclonal antibodies was detected by staining with Alexa Fluor 647-conjugated goat anti-human antibody (Jackson ImmunoResearch, Code: 109-605-097). Surface expression of CD4 was detected by staining with Brilliant Violet 421-conjugated anti-human CD4 antibody (BioLegend, clone: OKT4, cat: 317434). Data was collected using a BD FACSymphony A3 instrument and Env expression was assessed after gating on live, HIV-infected (eGFP+CD4low) cells.

### Antibody-dependent phagocytosis of fluorescent microspheres

ADP of fluorescent microspheres was measured as previously described [28]. HIV-1 JR-FL gp120 (Immune Technology, cat: IT-001-0024p) was biotinylated using EZ-Link Micro Sulfo-NHS-LC-Biotinylation Kit (Thermo Scientific, cat: 21935). The biotinylated HIV-1 gp41 MPER peptide (RRR-NEQELLELDKWASLWNWFDITNWLWYIR-RRK-biotin) was synthesized (GenScript) [27,73]. Biotinylated HIV-1 JR-FL gp120 and gp41 MPER peptide were incubated with yellow-green fluorescent NeutrAvidin-labeled microspheres (Invitrogen, cat: F8776) for 16 hours at 4°C. Optimal saturation conditions were determined experimentally. After coupling with biotinylated gp120, the beads were washed twice using 1% FACS buffer (PBS with 1% FBS) and resuspended 1:100 in 1% FACS buffer. 10 μl of this resuspension was then incubated with Env-specific monoclonal antibodies over a range of concentrations for 2 hours at 37°C in triplicate wells of round-bottom 96-well plates. THP-1 cells were added to each well (2 x 10^4^ cells/well) and incubated for 16 hours at 37°C. Following incubation, the cells were fixed in 2% paraformaldehyde PBS and analyzed by flow cytometry using a BD FACSymphony A3 instrument. Phagocytic score was calculated by multiplying the percentage of yellow-green fluorescent bead-positive single THP-1 cells by the mean fluorescence intensity (MFI) within the THP-1 cells and dividing the score by 1000 [76].

### ImageStream analysis

ADCP was corroborated using an ImageStream MarkII instrument. Images were captured for 2000 events in triplicate at each monoclonal antibody concentration. An internalization score was assigned to single PKH26+GFP+ THP-1 cells using the internalization feature of IDEAS software (Amnis version 6.3). Single PKH26+GFP+ THP-1 cells with positive scores were used to calculate percent internalization at each antibody concentration by dividing the total number of single PKH26+GFP+ THP-1 cells by the total number of single PKH26+ THP-1 cells.

### Live-cell and fixed-cell 3D confocal microscopy

mCherry THP-1 cells and HIV-1 NL4-3-infected SCR84 cells were co-cultured at a 1:1 E:T ratio and transferred to 8-well #1.5H glass bottom μ-slides (Ibidi). The cells were maintained at 37°C and 5% CO_2_ in a microscope stage-mounted live-cell culture chamber (Pathology Devices, Inc.). Live-cell imaging was performed using an A1R confocal microscope (Nikon) operated by NIS Elements Software (Nikon) and equipped with a Perfect Focus System (Nikon), motorized stage, 20X Plan Apo NA 0.75 objective lens, 488 nm and 560 nm excitation laser lines and a GaAsP PMT detection unit. Imaging was initiated within 15 minutes of co-culture and performed every minute for around 4 hours with an ORCA-Flash4.0 CMOS camera (Hamamatsu Photonics).

Fixed-cell 3D depictions were generated by co-culturing mCherry THP-1 cells and HIV-1 NL4-3-infected SCR84 in the presence of 0.5 μg/ml of PGT145 as described for ADCP assays. After 4.5 hours, co-cultures were adhered to 18 mm poly-d-lysine-coated glass coverslips (VWR, cat: 48380–046), fixed in 2% paraformaldehyde PBS, stained with Hoechst and mounted onto micro slides (VWR, cat: 48311–703) overnight. Z-stack images at 0.4 μm steps were captured using the microscope settings described above, apart from using a 100X Plan Apo NA 1.45 objective lens and the addition of a 405 nm excitation laser line. Images were processed and analyzed with FIJI/ImageJ [77].

### Statistical analysis

Statistical analyses were performed using GraphPad Prism v10.2.2. AUC values were calculated using a baseline of Y = 0 with peaks ignored that were less than 10% of the distance from minimum to maximum Y. Significant differences in AUC between antibodies were calculated by ordinary one-way ANOVA with Dunnett’s test using multiple comparisons by comparing each antibody to DEN3. ΔAUC and ΔGMFI values were calculated by subtracting the mean AUC and GMFI values of DEN3 from the mean AUC and GMFI values of Env-specific antibodies. Relationships were assessed using Spearman’s rank-order correlation coefficient.

## Supporting information

S1 Fig Gating strategy for measuring ADCP. Antibody-dependent phagocytosis of HIV-1 NL4-3-infected (eGFP+) SCR84 cells by PKH26-labeled THP-1 cells was determined by flow cytometry. An eGFP+ gate was set after gating on live, single PKH26+ THP-1 cells alone (A) and applied to HIV-1 NL4-3-infected SCR84 cells alone (B) and PKH26+ THP-1 cells incubated with HIV-1 NL4-3-infected SCR84 cells in the presence of PGT145 (0.5 μg/ml) (C).
(TIF)

S2 Fig A substitution in Env that interferes with CD4 binding diminishes ADCP by F240. (A) SCR84 cells were infected with an HIV-1 JR-FL mutant encoding a D368A substitution in Env that interferes with CD4 binding. On day 2 post-infection, infected SCR84 cells were incubated with PKH26-labeled THP-1 cells for 4.5 hours at a 1:3 E:T ratio in the presence of the indicated concentrations of PGT121 and F240. Phagocytic scores were calculated by multiplying the percentage of single PKH26+eGFP+ THP-1 cells by the GMFI of eGFP within the PKH26+THP-1 cell population. Error bars represent the standard deviation of the mean for triplicate wells. Antibody binding to Env on the surface of HIV-1-infected (eGFP+CD4low) SCR84 cells was confirmed by flow cytometry. Env staining was detected by staining with an AF647-conjugated goat anti-human antibody. (B) Mean AUC values were calculated from the phagocytic scores of duplicate assays and compared to the DEN3 control by ordinary one-way ANOVA with Dunnett’s test (ns, not significant, *p* > 0.05; *, *p* ≤ 0.05).
(TIF)

S1 File Experimental values. (XLSX)
