# Supplementary figures and images for: Potent broadly neutralizing antibodies mediate efficient antibody-dependent phagocytosis of HIV-infected cells
Source: PLoS Pathog. 2024 Oct 28;20(10):e1012665. doi: 10.1371/journal.ppat.1012665 (PMC11542898; doi:10.1371/journal.ppat.1012665)

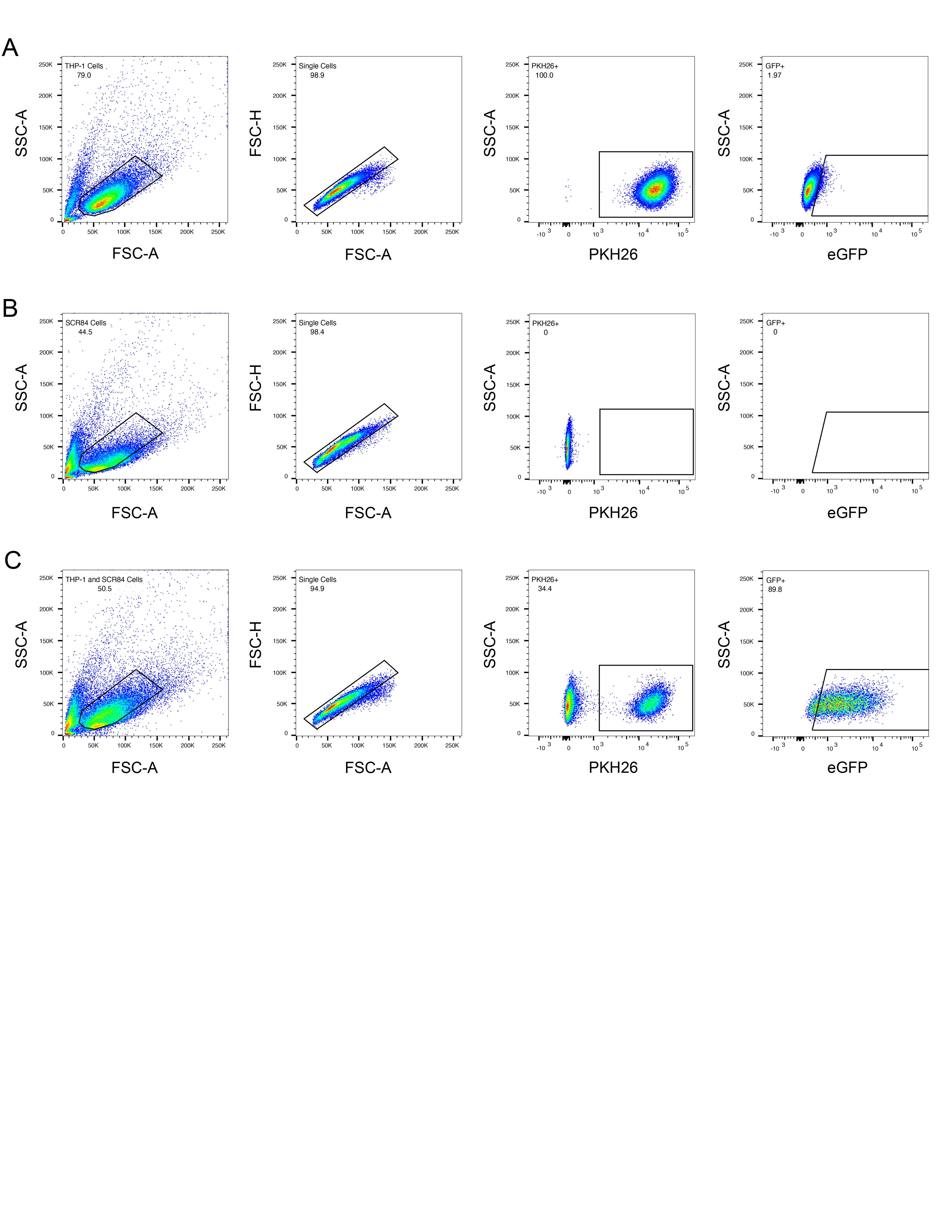

Supplement: S1 Fig — Antibody-dependent phagocytosis of HIV-1 NL4-3-infected (eGFP+) SCR84 cells by PKH26-labeled THP-1 cells was determined by flow cytometry. An eGFP+ gate was set after gating on live, single PKH26+ THP-1 cells alone (A) and applied to HIV-1 NL4-3-infected SCR84 cells alone (B) and PKH26+ THP-1 cells incubated with HIV-1 NL4-3-infected SCR84 cells in the presence of PGT145 (0.5 μg/ml) (C). (TIF) [file ppat.1012665.s001.tif]

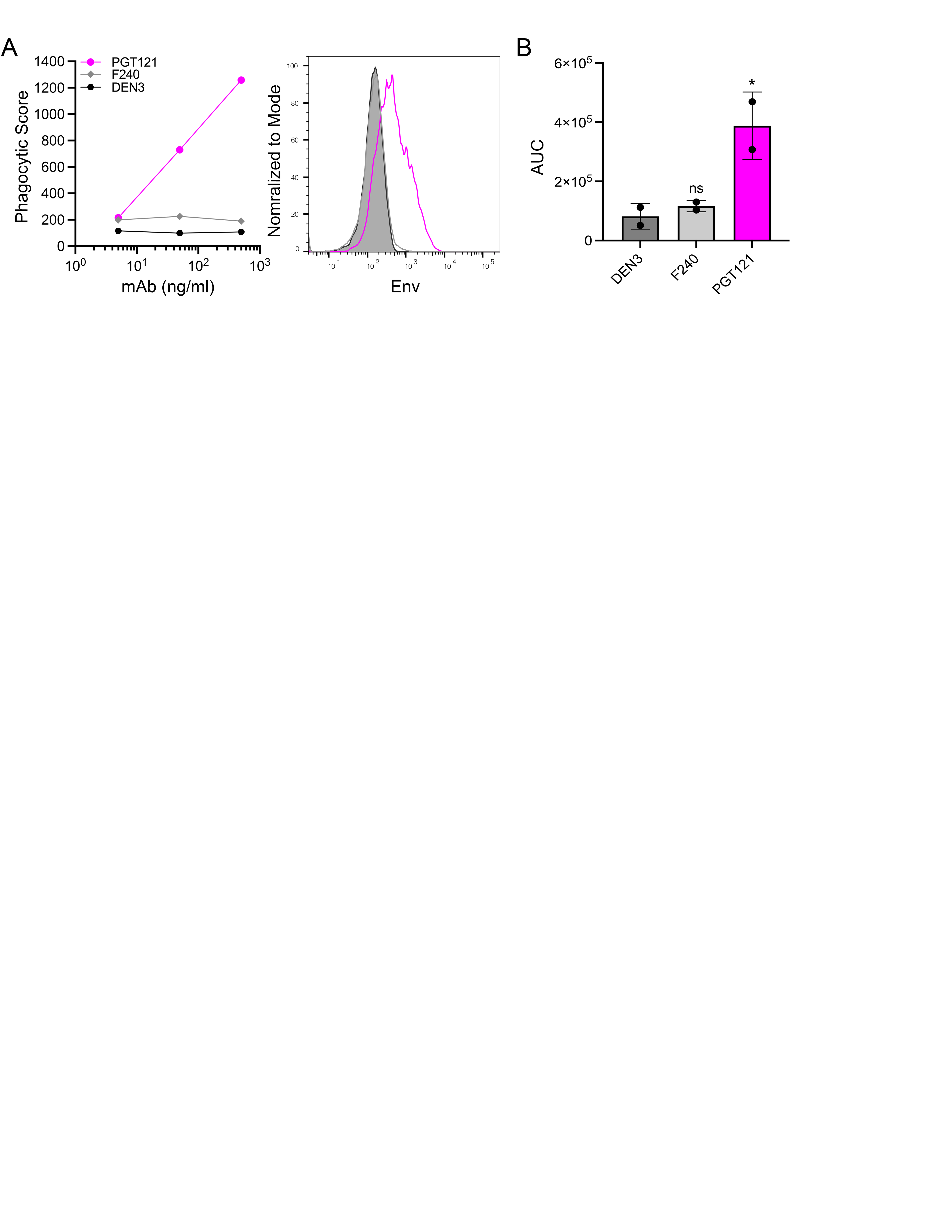

Supplement: S2 Fig — (A) SCR84 cells were infected with an HIV-1 JR-FL mutant encoding a D368A substitution in Env that interferes with CD4 binding. On day 2 post-infection, infected SCR84 cells were incubated with PKH26-labeled THP-1 cells for 4.5 hours at a 1:3 E:T ratio in the presence of the indicated concentrations of PGT121 and F240. Phagocytic scores were calculated by multiplying the percentage of single PKH26+eGFP+ THP-1 cells by the GMFI of eGFP within the PKH26+THP-1 cell population. Error bars represent the standard deviation of the mean for triplicate wells. Antibody binding to Env on the surface of HIV-1-infected (eGFP+CD4low) SCR84 cells was confirmed by flow cytometry. Env staining was detected by staining with an AF647-conjugated goat anti-human antibody. (B) Mean AUC values were calculated from the phagocytic scores of duplicate assays and compared to the DEN3 control by ordinary one-way ANOVA with Dunnett’s test (ns, not significant, p > 0.05; *, p ≤ 0.05). (TIF) [file ppat.1012665.s002.tif]
